# Zinc Detoxification: A Functional Genomics and Transcriptomics Analysis in *Drosophila melanogaster* Cultured Cells

**DOI:** 10.1534/g3.117.300447

**Published:** 2017-12-09

**Authors:** Stephanie E. Mohr, Kirstin Rudd, Yanhui Hu, Wei Roc Song, Quentin Gilly, Michael Buckner, Benjamin E. Housden, Colleen Kelley, Jonathan Zirin, Rong Tao, Gabriel Amador, Katarzyna Sierzputowska, Aram Comjean, Norbert Perrimon

**Affiliations:** * *Drosophila* RNAi Screening Center, Harvard Medical School, Boston, Massachusetts 02115; † Department of Genetics, Harvard Medical School, Boston, Massachusetts 02115; ‡ Howard Hughes Medical Institute, Boston, Massachusetts 02115

**Keywords:** metal detoxification, metal homeostasis, ABC transporters, glutathione, high-throughput screen

## Abstract

Cells require some metals, such as zinc and manganese, but excess levels of these metals can be toxic. As a result, cells have evolved complex mechanisms for maintaining metal homeostasis and surviving metal intoxication. Here, we present the results of a large-scale functional genomic screen in *Drosophila* cultured cells for modifiers of zinc chloride toxicity, together with transcriptomics data for wild-type or genetically zinc-sensitized cells challenged with mild zinc chloride supplementation. Altogether, we identified 47 genes for which knockdown conferred sensitivity or resistance to toxic zinc or manganese chloride treatment, and >1800 putative zinc-responsive genes. Analysis of the ‘omics data points to the relevance of ion transporters, glutathione (GSH)-related factors, and conserved disease-associated genes in zinc detoxification. Specific genes identified in the zinc screen include orthologs of human disease-associated genes CTNS, PTPRN (also known as IA-2), and ATP13A2 (also known as PARK9). We show that knockdown of *red dog mine (rdog*; *CG11897)*, a candidate zinc detoxification gene encoding an ABCC-type transporter family protein related to yeast cadmium factor (YCF1), confers sensitivity to zinc intoxication in cultured cells, and that *rdog* is transcriptionally upregulated in response to zinc stress. As there are many links between the biology of zinc and other metals and human health, the ‘omics data sets presented here provide a resource that will allow researchers to explore metal biology in the context of diverse health-relevant processes.

Whereas metals such as mercury or cadmium are solely toxic to cells, other metals, such as zinc and manganese, are essential for cell viability and are toxic only in excess. Zinc is a structural component of many proteins and is also thought to act as a signaling molecule (Fukada *et al.* 2011). Adding to the complexity of cellular zinc regulation, zinc is maintained at different levels in different organelles. Cells have evolved complex mechanisms for surviving zinc insufficiency, maintaining cellular and subcellular zinc homeostasis, and surviving exposure to toxic levels of zinc. The molecular mechanisms underlying regulation of zinc homeostasis and detoxification are, in some cases, zinc-specific, and in other cases relevant to other metals. Methods used by cells to maintain zinc levels and/or survive metal toxicity include the regulation of proteins required for metal influx (*e.g.*, ZIP family importers of zinc), metal efflux (*e.g.*, ZnT family exporters of zinc), or metal chelation (*e.g.*, by metallothioneins), as well as sequestration of zinc and/or biomolecules damaged by zinc in membrane-bound organelles, such as the yeast vacuole or mammalian lysosome (Kambe *et al.* 2015).

In addition to involving transporters and chelators, metal detoxification also involves more general detoxification strategies. GSH has long been known to have the ability to form a complex with zinc or cadmium (Perrin and Watt 1971). Data from plants, yeasts, tunicates, fish, and other organisms suggest the relevance of GSH levels, conjugation, and/or transport to metal detoxification (Perego and Howell 1997; Penninckx 2002; Gharieb and Gadd 2004; Franchi *et al.* 2012; Seth *et al.* 2012). Genetic evidence provides further support for a connection between GSH and metal detoxification. The ABCC-family transporter YCF1 is one example. YCF1 was originally identified based on a cadmium sensitivity phenotype and has been implicated in GSH-mediated detoxification of cadmium. The YCF1 protein is thought to be localized to the vacuole and to mediate transport of bis(glutathionato)cadmium into the vacuole (Nagy *et al.* 2006). Evidence suggests that the related protein YOR1, which is localized to the plasma membrane, can transport GSH-conjugated cadmium out of the cell. Consistent with this idea, *ycf1*, *yor1* double mutant yeast strains are reportedly more sensitive to cadmium intoxication than either single mutant strain (Nagy *et al.* 2006).

The biology of zinc and other metals has many connections with human diseases. For example, genetic disruption of genes encoding metal transporters can lead to diseases of metal insufficiency or excess (Clayton 2017). In addition, accumulation of high levels of metals, which can occur following consumption of contaminated drinking water or through occupational exposure, can negatively impact human development and cause disease. Further, because metals are used by cells as “weapons” in their defense against pathogens (Weiss and Carver 2017), metal insufficiency can impact immune function. Moreover, metals or metal-related genes have been implicated in, or levels correlated with, diseases such as diabetes, Parkinson’s disease, and Alzheimer’s disease (Chasapis *et al.* 2012). The zinc transporter ZNT8 is a common autoantigen in type 1 diabetes (Arvan *et al.* 2012), for example, and ATP13A2 (PARK9), a Parkinson’s disease gene, has been implicated in zinc homeostasis (Kong *et al.* 2014; Park *et al.* 2014; Tsunemi and Krainc 2014). Furthermore, adaptation of insect vectors of disease such as mosquitoes to metals might confer concomitant resistance to insecticides (Poupardin *et al.* 2008), such that understanding metal detoxification in insects might impact our understanding of disease vector control and the impact of polluted environments on the spread of insect-borne diseases (Poupardin *et al.* 2012).

Although yeast provides an excellent genetic platform for study of the cell biology of metal detoxification, using a single-celled organism has limited potential to model multicellular systems such as humans or insect vectors of disease. *Drosophila* presents many advantages as a genetic model system, including the study of metal homeostasis and detoxification at the cellular and whole-organism levels, study of the effects of genetic or environmental perturbation of metal levels in the context models of human diseases (*e.g.*, in *Drosophila* models of Parkinson’s or Alzheimer’s disease), and as a model of adaptation to metals and/or insecticides by insect vectors of disease. Work by several laboratories has established *Drosophila* as an *in vivo* model for study of zinc biology in a multicellular system (Richards and Burke 2016; Xiao and Zhou 2016), as well as for evolutionary studies of metal-related genes (Sadraie and Missirlis 2011; Rempoulakis *et al.* 2014). In particular, R. Burke and colleagues have performed a comprehensive genetic survey of *in vivo* ZIP and ZnT family zinc transporter functions using combined knockdown and overexpression approaches (Lye *et al.* 2012, 2013). Moreover, R. Burke, B. Zhou, and others have established the fly gut as a system for the study of zinc and other metals (Wang *et al.* 2009; Wang and Zhou 2010; Jones *et al.* 2015). Studies in *Drosophila* have also identified a role for zinc in kidney stone disease (Chi *et al.* 2015), and *Drosophila* is an established model in which to study the effects of metal-containing nanomaterials (Alaraby *et al.* 2016).

Altogether, the existing literature suggests that *Drosophila* provides an excellent system in which to study the cellular and organismal biology of metal homeostasis and detoxification. However, despite the growing body of work in *Drosophila* on zinc biology and other metal-related studies, there has remained a need for the application of high-throughput functional genomic methods for the study of zinc and other metals in *Drosophila*. Here, we describe the results obtained by applying two complementary ‘omics approaches to the identification of genes relevant to metal homeostasis and detoxification. Specifically, we performed large-scale *Drosophila* cell-based RNA interference (RNAi) screens to identify genes relevant to zinc or manganese detoxification, and performed a transcriptome-wide analysis of genes regulated in response to mild metal supplementation of wild-type or genetically zinc-sensitized cells. The results point to conserved genes and functions, and provide a resource for further study.

## Materials and Methods

### Cultured cell lines

The screen was performed using the *Drosophila* RNAi Screening Center (DRSC) isolate of the S2R+ *Drosophila* cell line. Derivatives of this cell line newly generated in this work are available from the *Drosophila* Genome Resource Center (DGRC) cultured cell repository in Bloomington, IN (DGRC cell IDs 1000 and 1001; see below and Supplementary Material, File S4 [Reagent Table]).

### Cell RNAi screening

In total, we screened four double-stranded RNA (dsRNA) reagent libraries for *Drosophila* cell-based RNAi screening from our DRSC collection (Hu *et al.* 2017b): the TM library targeting genes encoding transmembrane domain-containing proteins (17 unique 384-well assay plates), the AUTGY library targeting genes encoding autophagy-related factors (three plates), the MBO1 library targeting genes encoding proteins associated with membrane-bound organelles (two plates), and a custom-designed plate with candidate metal-related factors that we refer to as the “Megadeath” plate (one plate). In all cases, experimental dsRNAs were excluded from the outermost two wells of the final 384-well assay plate design to limit edge effects. Three replicates of each unique plate in the library (metal-supplemented conditions) or two replicates of each unique plate (control) were screened. To perform the screens, we added S2R+ cultured cells to dsRNA-containing assay plates as described previously (Echeverri and Perrimon 2006). We then incubated the plates in a 25° incubator with humidity control for 4 d. Next, freshly prepared ZnCl_2_ or MnCl_2_ (Sigma Aldrich) in solution or a control treatment (water) was added to the assay plates using a Formulatrix Mantis liquid handling robot to a final level of supplementation of 15 mM. Twenty-four hours following metal supplementation or control treatment, cells were lysed, and total ATP levels per well were determined using Promega Cell Titer Glo and a Molecular Devices Spectramax Paradigm luminometer. The step-by-step screen and assay protocols that we used are available online at https://fgr.hms.harvard.edu/fly-cell-rnai-384-well-format and https://fgr.hms.harvard.edu/fly-cell-total-atp-readout. Relative luciferase values for each plate were normalized to the plate average, replicates were averaged, and average normalized relative luciferase values were then compared across plates by calculating Z-scores.

### Generation of CRISPR knockout cell lines

The sgRNA sequence used to target *ZnT63C* was 5ʹ-TGTGACCAATTCGATGGCTC-3ʹ and the sgRNA sequence used to target IA-2 was 5ʹ-CGGCTGTTCCGCGTGCTCTCTGG-3ʹ (see also File S4). The sgRNAs were cloned and introduced into cells as described in Housden *et al.* (2014, 2015). Briefly, following the introduction of Cas9 and sgRNAs targeting *ZnT63C* or *IA-2* and single-cell cloning, we used high-resolution melt analysis (HRMA) to identify gene-modified cells. The *ZnT63C* or *IA-2* gene regions from colonies positive for HRMA were then amplified by PCR, PCR products were individually cloned by TOPO cloning, at least 10 isolates were subjected to Sanger sequencing, and sequence data were aligned and analyzed to confirm that all alleles contained frameshift mutations.

### RNA preparation and RNAseq analysis

For RNAseq analysis of control or metal-treated cells, wild-type S2R+ or CRISPR-modified mutant cell derivatives (*IA2-KO* and *ZnT63C-KO*) were first grown to confluency in 10 ml of media in T-75 flasks, control samples were next left untreated and experimental samples were supplemented to a final concentration of 1 mM ZnCl_2_ or MnCl_2_, and cells were then incubated for 24 hr, centrifuged, and resuspended in TRIzol reagent (Thermo Fisher). RNA was extracted as described previously using chloroform extraction and isopropanol precipitation (Song *et al.* 2010). Each RNA solution was divided into two aliquots, one of which was freshly used for RNA Integrity Number (RIN) evaluation at the Harvard Medical School Biopolymers Facility. If the RIN was >6.7 and no RNA degradation was observed following analysis, the other aliquot (stored at −80°) was shipped on dry ice to the Columbia Genome Center (CGC; Columbia University, New York, NY) for standard sample processing and raw data analysis. RNA libraries were prepared for sequencing using standard Illumina protocols and sequenced by Illumina HiSeq2000 at the CGC. The raw data files were analyzed by the CGC as follows. RTA (Illumina) was used for base calling and bcl2fastq (version 1.8.4) was used to convert BCL to fastq format, coupled with adaptor trimming. The reads were mapped to FlyBase genome annotation r5.51 using TopHat (version 2.0.4) with four mismatches and 10 maximum multiple hits. Relative abundance was determined using Cufflinks (version 2.0.2) with default settings and fragments per kilobase of transcript per million mapped reads (FPKM) values were calculated. The number of unique mapped reads per sample ranged from 17 million to 27 million. In order to compare the results at the gene level, we took the average of the FPKM values for each gene for the two replicates done for each condition, then determined the log_2_ ratio of FPKM levels for each genotype and treatment condition combination *vs.* FPKM levels in the same genotype. Prior to this analysis, we set to a value of “1” for any average FPKM value between 0 and 1 to reduce the possibility of getting large ratio values for genes with negligible levels of detected transcript in both the experimental sample and the wild-type control (*e.g.*, the ratio of FPKM 0.1 *vs.* 0.0001), as we assume those ratios are not likely to have biological relevance. A cutoff of twofold change for all replicates was applied (log_2_ > 1 or < −1).

### Real-time quantitative PCR (qPCR) analysis

Wild-type S2R+ cells were cultured under standard growth conditions in a 75 ml flask for 72 hr. Next, the appropriate amount of a 1 M stock solution of ZnCl_2_ or ZnSO_4_ (or water) was added to reach a final supplementation concentration of 0, 1, 3, or 5 mM. Cells were then incubated for 24 hr at 25°, centrifuged, resuspended in TRIzol (Thermo Fisher), treated with DNaseI, and cDNA was prepared using a QIAGEN RNeasy kit. The cDNA was analyzed by qPCR using SYBR Green and with tubulin as an internal reference gene control. All experiments were conducted in triplicate qPCR reactions. Data were analyzed with Bio-Rad CFX Manager software. Additional details regarding reagents and the sequence of oligonucleotide primer sequences used to detect experimental and control genes are indicated in the File S4.

### Enrichment analysis of the RNAi screen and RNAseq data sets

The gene hits from the RNAi screen and RNAseq profiling were analyzed for overrepresented gene sets using an in-house JAVA program based on hypergeometric distribution. The gene sets we queried were assembled using gene ontology (GO) annotation, pathway annotation from GLAD (Hu *et al.* 2015), and the *Drosophila* protein complex annotation from COMPLETE (Vinayagam *et al.* 2013). Human pathway annotation of Reactome (Croft *et al.* 2011) and KEGG (Kanehisa *et al.* 2017) were mapped to *Drosophila* gene sets using DIOPT (Hu *et al.* 2011), and included in enrichment analyses.

### Data availability

**Figure 1:**
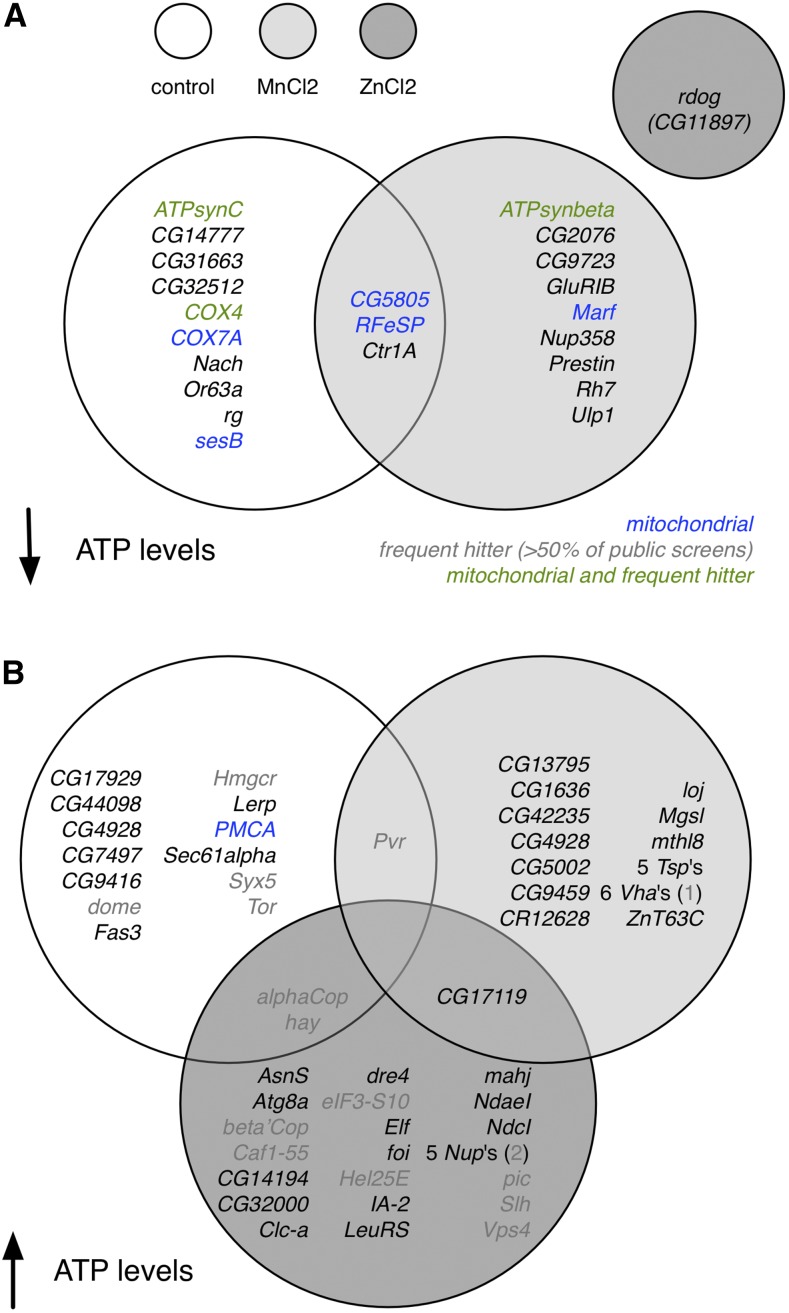
RNA interference screen hits for control and metal toxicity conditions. ATP levels down (A) or up (B). White circles, control conditions; light gray circles, MnCl_2_-supplemented conditions; dark gray circles, ZnCl_2_-supplemented conditions. Blue text, mitochondrial protein-encoding genes. Gray text, genes that frequently score as positives in other screens (>50% of public screens; frequent hitters). Green text, genes that are both mitochondrial-encoding and frequent hitters.

**Figure 2:**
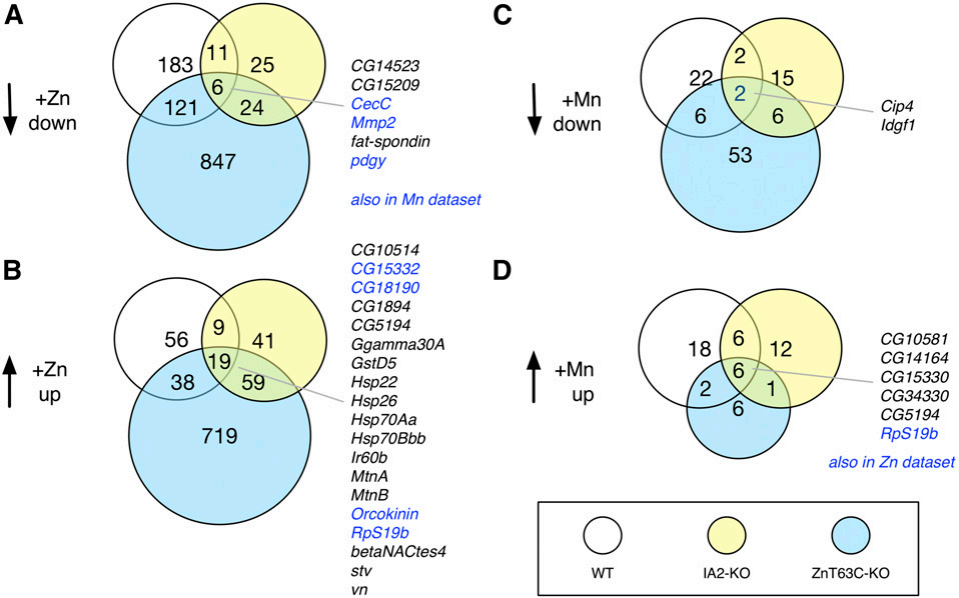
Overlap among genes down- or upregulated in wild-type (WT), *IA2-KO*, or *ZnT63C-KO* cells supplemented with zinc or manganese chloride. White circles, WT zinc-treated cells; yellow circles, *IA2-KO* zinc-treated cells; blue circles, *ZnT63C-KO* zinc-treated cells. (A and B) ZnCl_2_-treated conditions. Genes in common to all three genotypes for a given treatment condition are shown to the right. Blue text indicates genes also identified in the data set for one or more genotype treated with MnCl_2_. (C and D) MnCl_2_-treated conditions. Blue text indicates genes also identified in the data set for one or more genotype treated with ZnCl_2_ (the one gene that meets these criteria was common to all three zinc-treated genotypes). Fragments per kilobase of transcript per million mapped reads values for each of two replicates for each genotype and condition for the genes listed in the figure are provided in File S3.

High-confidence gene-level hits from the RNAi screens are shown in Figure 1 and listed with additional details (human orthologs, Z-scores, etc.) in Table 1, Table 2, and Table 3. In addition, a complete list of gene-level high-, moderate-, and low-confidence RNAi screen hits, as well as enrichment analysis results, is provided in File S1. Reagent-level RNAi screen data are available from the FlyRNAi database of the DRSC (see “Screen Summary” for a full list of screen data sets or “Gene Lookup” to query by gene or reagent) (Hu *et al.* 2017b). The RNAi screens were assigned DRSC Project IDs 177 through 185 and 193. To view the full data set for a screen, replace “X” with the three-digit DRSC Project ID in the URL http://www.flyrnai.org/cgi-bin/RNAi_public_screen.pl?project_id=X. The RNAi screen data sets are also available at NCBI PubChem BioAssay (Wang *et al.* 2014). The screens were assigned PubChem BioAssay IDs 1259314–1259316 and 1259326–1259331. To view a data set at PubChem BioAssay, replace “X” with the seven-digit PubChem ID in the URL https://pubchem.ncbi.nlm.nih.gov/bioassay/X. Analyzed results of the transcriptomics study are summarized in Figure 2 and Table 4, and provided in full in File S2 (gene-level data and enrichment analysis results). FPKM values for genes listed in Figure 2 or discussed are presented in File S3 (each of two replicates, all genotypes and conditions). In addition, the RNAseq data sets are available from the NCBI Gene Expression Omnibus, GEO accession ID GSE99332 (https://www.ncbi.nlm.nih.gov/geo/query/acc.cgi?acc=GSE99332).

**Table 1:** High-confidence RNAi screen results for control cells

FlyBase ID	Gene Symbol	Human Ortholog*^a^*	Treatment	Direction*^b^*	dsRNAs*^c^*	Avg Z-Score
FBgn0039830	*ATPsynC*	ATP5G2	Control	Down	2	−2.17
FBgn0026872	*CG14777*	MPV17L	Control	Down	2	−1.82
FBgn0051663	*CG31663*	–	Control	Down	2	−1.58
FBgn0052512	*CG32512*	TMEM205	Control	Down	2	−2.49
FBgn0039223	*CG5805*	SLC25A44	Control	Down	2	−1.97
FBgn0032833	*COX4*	COX4I1,2	Control	Down	2	−1.76
FBgn0040529	*COX7A*	COX7A2	Control	Down	2	−2.02
FBgn0062413	*Ctr1A*	SLC31A1	Control	Down	2	−2.06
FBgn0262743	*Fs(2)Ket*	KPNB1	Control	Down	2	−3.91
FBgn0024319	*Nach*	SCNN1B,G	Control	Down	2	−1.89
FBgn0035382	*Or63a*	–	Control	Down	2	−1.73
FBgn0021906	*RFeSP*	UQCRFS1	Control	Down	2	−2.93
FBgn0266098	*rg*	NBEA	Control	Down	2	−1.82
FBgn0003360	*sesB*	SLC25A4	Control	Down	2	−1.83
FBgn0025725	α*COP*	COPA	Control	Up	2	2.11
FBgn0038415	*CG17929*	–	Control	Up	2	2.17
FBgn0264907	*CG44098*	–	Control	Up	2	1.76
FBgn0027556	*CG4928*	UNC93A	Control	Up	2	1.99
FBgn0036742	*CG7497*	PTGER1,3,4	Control	Up	2	1.71
FBgn0034438	*CG9416*	ERMP1	Control	Up	2	1.85
FBgn0043903	*dome*	–	Control	Up	2	1.82
FBgn0000636	*Fas3*	–	Control	Up	2	2.97
FBgn0001179	*hay*	ERCC3	Control	Up	2	3.19
FBgn0263782	*Hmgcr*	HMGCR	Control	Up	2	2.59
FBgn0051072	*Lerp*	IGF2R	Control	Up	2	1.69
FBgn0259214	*PMCA*	ATP2B1,2,3,4	Control	Up	2	2.29
FBgn0032006	*Pvr*	FLT1	Control	Up	2	5.12
FBgn0086357	*Sec61*α	SEC61A1,2	Control	Up	2	2.48
FBgn0011708	*Syx5*	STX5	Control	Up	3	2.87
FBgn0021796	*Tor*	MTOR	Control	Up	2	2.51

ID, identifier; dsRNAs, double-stranded RNAs; Avg, average.

a Best match ortholog(s) are shown (DIOPT score cutoff > 2) (Hu *et al.* 2011).

b Down, decreased total ATP levels following plate-based normalization within a treatment group; up, increased total ATP levels following plate-based normalization within a treatment group.

c Number of unique dsRNAs in the library that target the gene. For all high-confidence hits as shown here, each of the designs resulted in a Z-score > 1.5 or < −1.5 and in the same direction as what was found for other designs targeting the same gene.

**Table 2:** High-confidence RNAi screen results for zinc chloride-treated cells

FlyBase ID	Gene Symbol	Human Ortholog(s)*^a^*	Treatment	Direction*^b^*	dsRNAs*^c^*	Avg Z-Score
FBgn0039644	*rdog*	ABCC family	ZnCl_2_	Down	2	−1.81
FBgn0025725	α*COP*	COPA	ZnCl_2_	Up	3	2.47
FBgn0270926	*AsnS*	ASNS	ZnCl_2_	Up	2	1.85
FBgn0052672	*Atg8a*	GABARAP	ZnCl_2_	Up	2	2.20
FBgn0025724	β’*COP*	COPB2	ZnCl_2_	Up	3	2.54
FBgn0263979	*Caf1-55*	RBBP4,7	ZnCl_2_	Up	2	2.09
FBgn0030996	*CG14194*	TMEM185A,B	ZnCl_2_	Up	2	3.22
FBgn0039045	*CG17119*	CTNS	ZnCl_2_	Up	2	4.88
FBgn0052000	*CG32000*	ATP13A2,3,4,5	ZnCl_2_	Up	3	4.14
FBgn0051116	*ClC-a*	CLCN1,2	ZnCl_2_	Up	2	3.04
FBgn0002183	*dre4*	SUPT16H	ZnCl_2_	Up	3	3.30
FBgn0037249	*eIF3-S10*	EIF3A	ZnCl_2_	Up	3	2.22
FBgn0020443	*Elf*	GSPT1,2	ZnCl_2_	Up	2	1.72
FBgn0024236	*foi*	SLC39A family	ZnCl_2_	Up	2	3.42
FBgn0001179	*hay*	ERCC3	ZnCl_2_	Up	2	3.21
FBgn0014189	*Hel25E*	DDX39A,B	ZnCl_2_	Up	3	7.74
FBgn0031294	*IA-2*	PTPRN,N2	ZnCl_2_	Up	2	2.54
FBgn0284253	*LeuRS*	LARS, LARS2	ZnCl_2_	Up	2	1.73
FBgn0034641	*mahj*	DCAF1	ZnCl_2_	Up	3	1.87
FBgn0259111	*Ndae1*	SLC4A family	ZnCl_2_	Up	2	1.60
FBgn0039125	*Ndc1*	NDC1	ZnCl_2_	Up	2	1.87
FBgn0039004	*Nup133*	NUP133	ZnCl_2_	Up	2	3.01
FBgn0021761	*Nup154*	NUP155	ZnCl_2_	Up	3	2.69
FBgn0039302	*Nup358*	RANBP2	ZnCl_2_	Up	2	3.17
FBgn0027537	*Nup93-1*	NUP93	ZnCl_2_	Up	2	5.60
FBgn0039120	*Nup98-96*	NUP98	ZnCl_2_	Up	2	6.02
FBgn0260962	*pic*	DDB1	ZnCl_2_	Up	3	3.22
FBgn0264978	*Slh*	SCFD1	ZnCl_2_	Up	2	1.58
FBgn0283469	*Vps4*	VPS4A,B	ZnCl_2_	Up	2	4.60

ID, identifier; dsRNAs, double-stranded RNAs; Avg, average.

a Best match ortholog(s) or paralog families are shown (DIOPT score cutoff > 2) (Hu *et al.* 2011).

b Down, decreased total ATP levels following plate-based normalization within a treatment group; up, increased total ATP levels following plate-based normalization within a treatment group.

c Number of unique dsRNAs in the library that target the gene. For all high-confidence hits as shown here, all of the designs resulted in a hit as defined by a Z-score > 1.5 or < −1.5, and in the same direction as what was found for other designs targeting the same gene.

**Table 3:** High-confidence RNAi screen results for manganese chloride-treated cells

FlyBase ID	Gene Symbol	Human Ortholog*^a^*	Treatment	Direction*^b^*	dsRNAs*^c^*	Avg Z-Score
FBgn0010217	*ATPsyn*β	ATP5B	MnCl_2_	Down	2	−2.45
FBgn0030263	*CG2076*	GHITM	MnCl_2_	Down	2	−1.63
FBgn0039223	*CG5805*	SLC25A44	MnCl_2_	Down	2	−1.80
FBgn0030768	*CG9723*	NEMP1,2	MnCl_2_	Down	2	−1.79
FBgn0062413	*Ctr1A*	SLC31A1	MnCl_2_	Down	3	−1.90
FBgn0264000	*GluRIB*	GRIA1,2,3,4	MnCl_2_	Down	2	−1.57
FBgn0029870	*Marf*	MFN2	MnCl_2_	Down	2	−2.81
FBgn0039302	*Nup358*	RANBP2	MnCl_2_	Down	2	−2.17
FBgn0036770	*Prestin*	SLC26A5	MnCl_2_	Down	2	−2.46
FBgn0021906	*RFeSP*	UQCRFS1	MnCl_2_	Down	2	−2.74
FBgn0036260	*Rh7*	OPN4,3	MnCl_2_	Down	2	−2.09
FBgn0027603	*Ulp1*	SENP1,2,3,5	MnCl_2_	Down	2	−1.83
FBgn0031937	*CG13795*	–	MnCl_2_	Up	2	3.32
FBgn0030030	*CG1636*	–	MnCl_2_	Up	2	3.20
FBgn0039045	*CG17119*	CTNS	MnCl_2_	Up	2	2.38
FBgn0250757	*CG42235*	SLC5A family	MnCl_2_	Up	3	2.92
FBgn0027556	*CG4928*	UNC93A	MnCl_2_	Up	2	1.71
FBgn0034275	*CG5002*	SLC26A11	MnCl_2_	Up	2	1.81
FBgn0037764	*CG9459*	ELOVL7	MnCl_2_	Up	2	2.36
FBgn0042701	*CR12628*	–	MnCl_2_	Up	2	3.10
FBgn0061492	*loj*	TMED6	MnCl_2_	Up	2	1.95
FBgn0025814	*Mgstl*	MGST1	MnCl_2_	Up	2	3.10
FBgn0052475	*mthl8*	–	MnCl_2_	Up	2	2.38
FBgn0032006	*Pvr*	FLT1	MnCl_2_	Up	2	3.46
FBgn0031760	*Tsp26A*	TSPAN5	MnCl_2_	Up	2	1.99
FBgn0029508	*Tsp42Ea*	CD63	MnCl_2_	Up	2	2.92
FBgn0033136	*Tsp42Eo*	–	MnCl_2_	Up	2	2.59
FBgn0033137	*Tsp42Ep*	–	MnCl_2_	Up	2	2.37
FBgn0033139	*Tsp42Er*	–	MnCl_2_	Up	2	1.98
FBgn0022097	*Vha36-1*	ATP6V1D	MnCl_2_	Up	2	1.64
FBgn0040377	*Vha36-3*	ATP6V1D	MnCl_2_	Up	2	2.21
FBgn0262511	*Vha44*	ATP6V1C1	MnCl_2_	Up	2	2.05
FBgn0263598	*Vha68-2*	ATP6V1A	MnCl_2_	Up	2	2.57
FBgn0028662	*VhaPPA1-1*	ATP6V0B	MnCl_2_	Up	2	1.99
FBgn0027779	*VhaSFD*	ATP6V1H	MnCl_2_	Up	3	2.37
FBgn0035432	*ZnT63C*	SLC30A1	MnCl_2_	Up	2	2.27

ID, identifier; dsRNAs, double-stranded RNAs; Avg, average.

a Best match ortholog(s) or paralog families are shown (DIOPT score cutoff > 2) (Hu *et al.* 2011).

b Down, decreased total ATP levels following plate-based normalization within a treatment group; up, increased total ATP levels following plate-based normalization within a treatment group.

c Number of unique dsRNAs in the library that target the gene. For all high-confidence hits as shown here, all of the designs resulted in a “hit” as defined by a Z-score > 1.5 or < −1.5 and in the same direction as what was found for other designs targeting the same gene.

**Table 4:** Summary of transcriptomics analysis of wild-type and zinc-sensitized cells under control or metal-supplemented conditions

	Wild-Type S2R+	*IA2-KO*	*ZnT63C-KO*
+ 1 mM ZnCl_2_	319 down	66 down	998 down
	121 up	128 up	835 up
+ 1 mM MnCl_2_	23 down	26 down	68 down
	33 up	26 up	16 up

Down, downregulated as compared with untreated cells of the same genotype; up, upregulated as compared with untreated cells of the same genotype.

## Results and Discussion

### Drosophila cell-based screen for modifiers of metal chloride toxicity

We reasoned that the application of high-throughput ‘omics approaches in *Drosophila* cultured cells would provide a robust data set that can inform future *in vivo* studies. To get a genome-scale view of genes relevant to zinc detoxification, we performed three large-scale RNAi screens in parallel using *Drosophila* S2R+ cultured cells. We used total ATP levels as an indirect assay readout of cell viability and number, and screened under three conditions: control conditions, toxic zinc conditions (*i.e.*, supplementation to a concentration of 15 mM ZnCl_2_), and toxic manganese conditions (15 mM MnCl_2_). Although our main goal was to identify genes relevant to zinc, we chose to screen in parallel under toxic manganese conditions for two reasons. First, we reasoned that screening with an equimolar concentration of a different metal chloride salt would help exclude the possibility that the genes we identify in the zinc intoxication screen are relevant to the chloride ion, changes in osmolarity, or other nonspecific effects. Second, we reasoned that the data would provide a helpful high-throughput data set relevant to the biology of manganese, a metal that, like zinc, is essential for cell viability and toxic in excess, but underexplored in the literature.

In order to quickly identify high-confidence screen hits (positive results) at the gene level, we screened RNAi reagent libraries from the DRSC collection that have multiple unique RNAi reagents per gene coverage and optimized layouts. Specifically, we screened four libraries of dsRNAs: a large library targeting known or predicted *Drosophila* transmembrane domain-containing protein-encoding genes and three smaller libraries targeting genes encoding putative autophagy-related factors (ZnCl_2_ conditions only), conserved components of membrane-bound organelles, or candidate metal-related factors (total of 3885 dsRNAs targeting 1960 unique genes). Each screen condition was internally normalized (see *Materials and Methods*). How to access screen data is described in the *Data availability* section of the *Materials and Methods*.

Several indicators suggest that the screens resulted in high-quality, metal-specific results. First, consistent with the identification of condition-specific results, replicate assay plates within a treatment condition correlate well (Pearson correlation coefficients of 0.88–0.93) but replicate assay plates from different treatment groups do not (Pearson correlation coefficients of 0.24–0.50). Second, for many genes, two unique reagents targeting the same gene scored as hits under the same conditions and in the same direction. We define this set of genes for which two unique reagents scored in the same direction in a given condition as the set of high-confidence hits (Figure 1, Table 1, Table 2, and Table 3). Third, the set of high-confidence hits in any given condition and direction include multiple members of a protein family (*e.g.*, several tetraspanin family proteins are high-confidence hits in the same direction in the manganese screen), multiple members of a protein complex (*e.g.*, several nuclear pore components are high-confidence hits in the same direction in the zinc screen), or multiple components of a given organelle (*e.g.*, for both control and manganese conditions, mitochondrial proteins are among the high-confidence hits with lower ATP levels *vs.* the internal controls; Figure 1A, blue or green text).

Altogether, we identified 30 high-confidence hits in the control treatment group, 29 in the zinc toxicity treatment group, and 36 in the manganese toxicity treatment group (Figure 1, Table 1, Table 2, and Table 3). Hits were identified from all four libraries, with 23% of genes in the candidate metal-related gene library identified as high-confidence hits and 4–8% of genes in the other three libraries identified as high-confidence hits. There is limited overlap among genes that scored as high-confidence hits from each of the three screens. Three genes (*CG5805*, *Ctr1A*, and *RFeSP*) are in common between high-confidence hits for control conditions, decreased ATP (“down”) direction and MnCl_2_ conditions, down direction (Figure 1A). Two genes (α*COP*, *hay*) are in common between high-confidence hits for control conditions, increased ATP (“up”) direction and ZnCl_2_-supplemented conditions, up direction; one gene (*Pvr*) is in common between high-confidence hits for control conditions, up direction and MnCl_2_-supplemented conditions, up direction; and one gene (*CG17119*) is in common between high-confidence hits for ZnCl_2_-supplemented conditions, up direction and MnCl_2_-supplemented conditions, up direction (Figure 1B). For the zinc toxicity screen, there was a clear bias in detection of high-confidence hits conferring higher ATP levels (28 high-confidence hits) as compared with lower ATP levels (one high-confidence hit). We suspect that the zinc treatment conditions were so toxic that it was difficult to detect a significant further reduction in total ATP levels *vs.* the internal control. In addition, some genes identified in the screens are “frequent hitters” (Figure 1, gray or green text), which we define here as hits scoring in >50% of *Drosophila* RNAi screens in the GenomeRNAi database (Schmidt *et al.* 2013).

### False discovery analysis

We used two approaches to address false positive and false negative discovery in the RNAi screens. As a first approach, we used RNAseq data from the modENCODE project (modEncode Consortium *et al.* 2010; Hu *et al.* 2017a) and this study (below) to determine the fraction of hits for which there is no evidence of expression in S2R+ cells (FPKM < 1), suggesting false positive discovery. This was true for 16 of 86 high-confidence hits (19%), 33 of 164 moderate-confidence hits (20%), and 246 of 548 low-confidence hits (45%), suggesting a false positive discovery rate of ∼20% for high- and moderate-confidence hits in the screens. Notably, the zinc screen appears to contribute the least to false positive discovery: only 2 of 29 high-confidence hits in the ZnCl_2_-supplemented conditions screen (7%) have FPKM < 1. Moreover, these two genes are annotated as having zinc or chloride ion-related functions. Thus, the false positive discovery rate for the zinc screen appears to be low.

Our second approach to false discovery analysis was to compare the results of the control screen to a previously published RNAi screen reported by Boutros *et al.* (2004) for genes essential in *Drosophila* cultured cells (Boutros *et al.* 2004). Among the genes identified as essential in that study, 11 met the criteria that they were (a) identified using dsRNA designs that meet current cutoffs for quality and (b) included in our control conditions screen. For 2 of 11, the FPKM values for cultured cell lines are <1, suggesting that these are false positive results in the Boutros *et al.* (2004) study. Of the nine genes with evidence for expression in cultured cells, three of nine scored in the down direction in our control screen (*Nup153* and *Desat1*, low-confidence hits and *Fs(2)Ket*, high-confidence hit), consistent with the idea that these are essential genes in S2R+ cells. We next asked if human cancer cell essential gene data support the idea that the six of nine genes identified in the Boutros screen and expressed but not identified in our control screen are indeed essential genes. We identified a set of human cancer cell essential genes based on two studies that together interrogated 11 different cell lines (Hart *et al.* 2015; Wang *et al.* 2015), then used DIOPT (Hu *et al.* 2011) to define orthologs and compared the gene lists. An ortholog of *ATPsyn*β is among the set of high-confidence human cancer cell essential genes (scored as essential in 8 of 11 human cell lines). Orthologs of *crb*, *crq*, and *Pvr* were not identified as essential in any of the cell lines; a *Kap*-α*3* ortholog was identified in 3 of 11 cell lines; and an *ACC* ortholog in 5 of 11 cell lines. Taken together, these data suggest that *crb*, *crq*, *Kap*-α*3*, and *Pvr* are likely to be false positives in the Boutros cell screen, and that *ATPsyn*β and *ACC* are likely to be false negatives in our control conditions screen. We note that *Nup153*, *Desat1*, *Fs(2)Ket*, and *ATPsyn*β were all hits in the down direction under MnCl_2_ supplementation conditions. We are reluctant to assign a false negative rate based on this analysis because only 11 genes could be compared.

### Analysis of zinc screen results

We found a number of zinc-related genes among the high-confidence hits in the zinc screen. For example, *fear of intimacy (foi)*, which encodes a ZIP family zinc influx protein (Mathews *et al.* 2005), was identified as a high-confidence hit for increased total ATP levels (up hits) in the zinc screen but not in the other screens (Table 2). The zinc screen hits in the up direction also include *CG32000*, a putative ortholog of human ATP13A2 (PARK9); evidence from mammalian cells suggests a role for ATP13A2 in lysosomal zinc transport (Kong *et al.* 2014; Park *et al.* 2014; Tsunemi and Krainc 2014). The zinc screen hits in this direction further include *IA-2 protein tyrosine phosphatase* *(IA-2)*, an ortholog of human PTPRN (better known as IA2), which, like the human zinc transporter ZNT8, is a common autoantigen associated with type 1 diabetes (Arvan *et al.* 2012).

The single high-confidence hit in the lower ATP levels direction in the zinc screen (*i.e.*, knockdown appears to confer zinc sensitivity; down hits) is a gene with the systematic name *CG11897*, which we have renamed *red dog mine (rdog)* after the large Alaskan zinc mine of that name. The *rdog* gene encodes a member of the ABCC subfamily of ABC transporters that also includes yeast YCF1. Five additional genes were initially categorized as low-confidence hits in the zinc treatment, lower ATP levels (down) category: *CG7627*, *CG3790*, *COX7AL*, *CR43469*, and *mthl3* (File S1). Notably, like *rdog*, *CG7627* also encodes an ABCC family member, further supporting the idea that ABCC-type transporters are relevant to zinc chloride detoxification. Based on enrichment analysis (below), *CG7627* was promoted to a moderate-confidence hit (File S1).

We performed enrichment analysis for GO “cellular compartment” or “biological process” terms, pathways as annotated in Reactome (Croft *et al.* 2011), and protein complexes as annotated by COMPLETE (Vinayagam *et al.* 2013). In all cases, we used the full set of hits in the analysis (*i.e.*, both low- and high-confidence hits). We reannotated low-confidence hits as moderate confidence if they were members of a significant enrichment group (File S1). Overall, the results of the enrichment analysis further suggest the quality and specificity of the screens. For the zinc toxicity screen, enrichment is driven by the presence of multiple components of the nuclear pore complex (*e.g.*, enrichment for GO cellular compartment “nuclear pore,” p-value 7.15e^−07^), suggesting the possible involvement of nuclear transport in zinc-induced cell death or another relevant process. We also note that there are related genes in the human genome for all of the high-confidence hits identified in the zinc screen (human orthologs are included in Table 2).

### Transcriptomics analysis of wild-type and zinc-sensitized cells

The use of genetically zinc-sensitized strains of *Drosophila* has helped uncover mechanisms of zinc homeostasis *in vivo* (Lye *et al.* 2012, 2013). We reasoned that production of mutant cell lines lacking activity of the zinc exporter *ZnT63C* would similarly result in cultured cells genetically sensitized to zinc supplementation and allow for detection of zinc-related genes. Such an approach would allow us to capitalize on the advantages of performing transcriptomics studies in a relatively homogenous cultured cell line, allowing for robust detection of down- or upregulated genes, as well as minimize the need to treat cells with high-ionic strength solutions. We used a CRISPR-Cas9 strategy to target *ZnT63C*, which encodes a zinc efflux protein, and also targeted *IA-2*, which encodes the *Drosophila* ortholog of human PTPRN/IA2, which, like the human zinc transporter ZNT8, is a common autoantigen in type 1 diabetes (Arvan *et al.* 2012). Following transfection of CRISPR reagents, single-cell isolation, and identification of candidate knockout cell lines (see *Materials and Methods*), we confirmed by Sanger sequencing the presence of frameshift mutations in all copies of the genes in clonal cell lines. We will refer to the cell lines hereafter as *ZnT63-KO* and *IA2-KO*.

We next treated wild-type S2R+, *IA2-KO*, or *ZnT63-KO* cells with mild zinc or manganese chloride supplementation, and isolated RNA from the samples for next-generation transcriptome sequencing (RNAseq). In total, we performed RNAseq analysis on two replicates each of nine combinations of genetic and treatment conditions: wild-type, *IA2-KO*, or *ZnT63C-KO* cells under control, mild zinc supplementation (1 mM ZnCl_2_), or mild manganese supplementation conditions (1 mM MnCl_2_). Following sequencing, we obtained analyzed FPKM values and used these as the data set for all subsequent analyses. The number of down- or upregulated genes for mild zinc and manganese chloride supplementation conditions, relative to values for untreated cells of the same genotype, is summarized in Table 4. We observed a larger overall transcriptional response in *ZnT63C-KO* cells treated with ZnCl_2_ than in the other genotypes and conditions (*i.e.*, a larger total number of genes with significant log_2_ values as compared to the control and a large fold-change among the top hits), consistent with the idea that *ZnT63C-KO* are more sensitive to zinc supplementation than wild-type S2R+ cells. File S2 includes a list of the genes the data suggest are down- or upregulated in each genotype and condition. File S3 displays the read counts for each replicate for genes listed in Figure 2 or discussed. How to access the RNAseq data is outlined in the *Data availability* section of the *Materials and Methods*.

**Figure 3:**
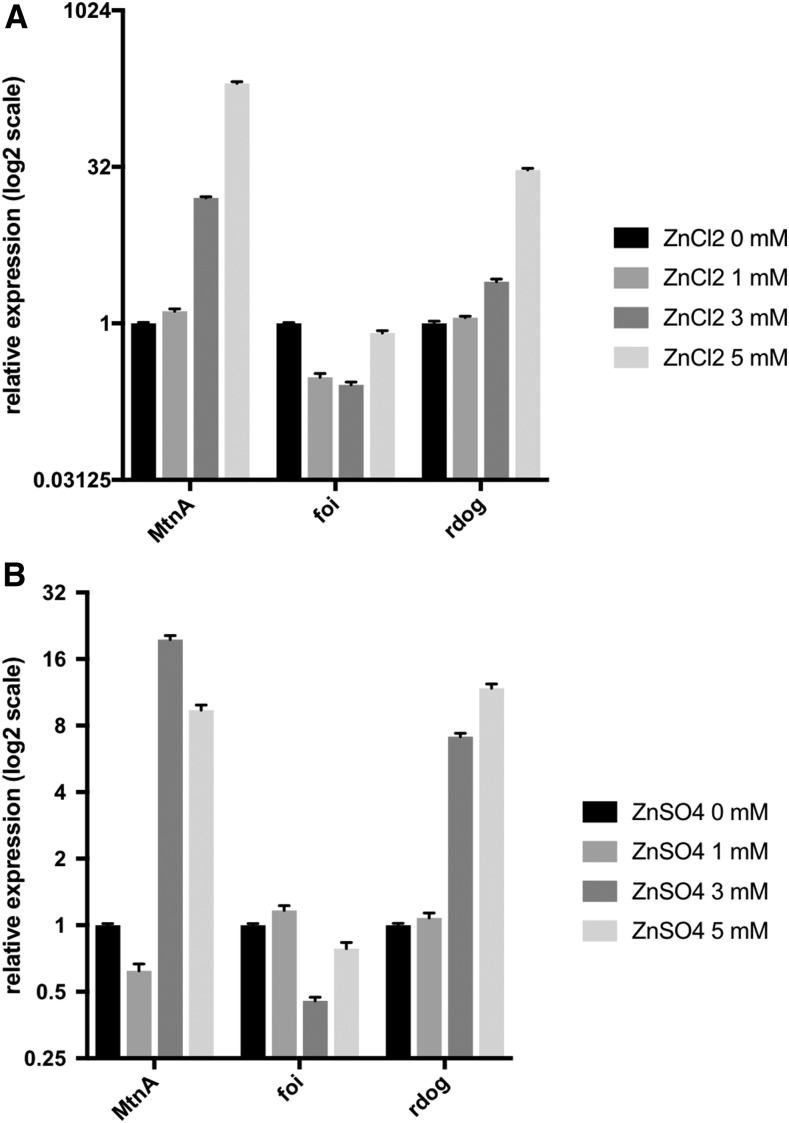
The ABCC-family transporter *rdog* is upregulated in *Drosophila* S2R+ cells in response to zinc treatment. (A) Quantitative polymerase chain reaction (qPCR) analysis of control and *rdog* transcript levels in S2R+ cells supplemented with ZnCl_2_ to a final concentration of 0, 1, 3, or 5 mM. The analyzed data are shown on a log_2_ scale. RNA levels from metal treatment samples were normalized to the 0 mM control (see *Materials and Methods*). Error bars indicate SEM. (B) qPCR analysis of control and *rdog* transcript levels in S2R+ cells supplemented with ZnSO_4_ to a final concentration of 0, 1, 3, or 5 mM. The analyzed data are shown on a log_2_ scale. Consistent with a response to zinc, *rdog* transcript levels are upregulated in response to zinc sulfate supplementation. RNA levels from metal treatment samples were normalized to the 0 mM control (see *Materials and Methods*). Error bars indicate SEM.

As summarized in Table 4, we identified >1800 putative zinc-responsive genes. Several indicators suggest that the strategy of genetically sensitizing cells to zinc supplementation was successful in identifying high-confidence zinc-responsive genes, including the observation that several genes were down- or upregulated in two or more zinc-supplemented S2R+, *IA2-KO*, and/or *ZnT63C-KO* genotypes (Figure 2). Not surprisingly, the set of zinc-responsive genes includes metallothioneins, which act as metal chelators, as well as a number of heat-shock proteins (Figure 2B and Table S1). Interestingly, at least for some genes, the level of upregulation induced by zinc in *IA2-KO* cells was intermediate as compared with wild-type and *ZnT63C-KO* zinc-treated cells (File S2 and Table S1). As compared with zinc, supplementation with low levels of MnCl_2_ of any genotype resulted in a small number of genes showing significant down- or upregulation as compared to wild-type untreated cells (Figure 3, File S2, and Table 4), consistent with the idea that knockout of *ZnT63C* sensitizes cells to zinc but not manganese. However, despite the smaller numbers, some genes are in common between the zinc and manganese data sets (Figure 2 and File S2), suggesting that these genes might be chloride-responsive or general factors. The knockout cell production method that we used includes a single-cell cloning step. Single-cell cloning alone could, in principle, result in changes that affect the transcriptional profile. For this reason, we consider the highest confidence zinc-responsive genes to be those found in all three zinc-treated genotypes. These are listed in Figure 2.

### Enrichment analysis of transcriptomics data from zinc-treated cells

As we did with the RNAi screen data, we performed enrichment analysis of the transcriptomics data to detect GO terms, molecular pathways, or protein complexes that were significantly enriched in these data sets (File S2). Enrichment among genes downregulated in response to ZnCl_2_ supplementation includes processes or protein complexes related to ribosomes (*e.g.*, Reactome “translation,” p-value 6.88 × 10^−15^ in zinc-treated wild-type cells), and pathways or cellular components of respiratory electron transport. Enrichment among genes upregulated in response to ZnCl_2_ supplementation includes processes or complexes involving heat-shock proteins and *starvin* (*stv)*, an ortholog of the human “BCL2-associated athanogene 3” or BAG3 gene. Significant enrichment was also seen in zinc-treated wild-type cells for the GO molecular function heme-copper terminal oxidase activity; 3 of the 21 genes in this group that result in enrichment are *COX4L* and *COX7AL*, which encode subunits of cytochrome c oxidase, and *CG42376*, which encodes an ortholog of human “cytochrome c oxidase assembly factor 6” or COA6. For zinc-treated *ZnT63C-KO* cells, upregulated genes are also enriched for GSH-related activities (*e.g.*, KEGG “glutathione metabolism,” p-value 3.62 × 10^−9^ in *ZnT63-KO* cells). Indeed, 9 of 11 GstD subfamily genes, other GSH S-transferase genes, and *Glutamate-cysteine ligase catalytic subunit* *(Gclc)*, a rate-limiting enzyme in the GSH synthesis pathway, are upregulated in zinc-treated *ZnT63C-KO* cells.

Altogether, the transcriptomics data show that zinc stress results in upregulation of metal chelators and heat-shock proteins, and suggests that zinc stress has specific impacts on mitochondrial function that elicit compensatory transcriptional responses. Moreover, the data obtained using a zinc-sensitized genotype suggest that, under high zinc stress conditions, there is a significant need for conjugation of substrates to GSH. This is consistent with a recent report that GSH S-transferase activity is relevant to methyl mercury toxicity in *Drosophila* (Vorojeikina *et al.* 2017), and with results obtained for other species that associate metal detoxification with GSH conjugation and/or flux (Perego and Howell 1997; Penninckx 2002; Gharieb and Gadd 2004; Nagy *et al.* 2006; Franchi *et al.* 2012; Seth *et al.* 2012).

### Comparison of functional screen and transcriptomics data

We reasoned that genes encoding proteins normally involved in zinc influx would be expected to score in the up direction in the screen (higher ATP values as compared with the internal control, consistent with resistance to zinc treatment) and down in response to zinc supplementation in the transcriptomics analysis. Consistent with this, we found that the high-confidence screen hit *foi* was downregulated in zinc-treated S2R+ and zinc-treated *IA2-KO* cells as compared with untreated cells of the same genotype, and downregulated in zinc-treated *ZnT63C-KO* cells as compared with wild-type untreated cells. In addition, *CG32000* was downregulated in zinc-treated *ZnT63C-KO* cells as compared with wild-type untreated cells. We also compared the data for components of the nuclear pore. The low-confidence screen hit *Nup107* was downregulated in zinc-treated wild-type and zinc-treated *ZnT63C-KO* cells as compared with genotype controls, and in zinc-treated *IA2-KO* cells as compared with untreated cells of the same genotype. In addition, the high-confidence hit *Nup93-1* was downregulated in zinc-treated *ZnT63C-KO* cells as compared with the wild-type untreated control, and the additional nuclear pore component-encoding genes *Nup43*, *Nup44A*, *Nup50*, *Nup54*, and *Nup160* were downregulated in *ZnT63C-KO* cells as compared with untreated cells of the same genotype.

We next explored the converse prediction: that genes encoding proteins protective against zinc intoxication would be expected to score in the down direction in the screen and to be upregulated in response to zinc supplementation. Despite the relatively small number of down direction hits in the zinc screen (Figure 1, File S1, and Table 1), we did find overlap between the RNAi down direction hits and zinc-responsive gene lists. The low-confidence hit *COX7AL* was significantly upregulated in zinc-treated S2R+ and zinc-treated *ZnT63C-KO* cells as compared with untreated genotype controls, and in all three as compared with untreated wild-type cells. In addition, the one high-confidence down direction RNAi screen hit, *rdog*, scored as significantly upregulated in zinc-treated *ZnT63C-KO* cells; the log_2_ values for *rdog* were 1.21 for zinc-treated *IA2-KO* cells and 3.53 for zinc-treated *ZnT63C-KO* cells as compared with genotype controls (FPKM values for each of two replicates in all genotypes and conditions are provided in File S3).

### rdog is upregulated in response to zinc in Drosophila S2R+ cells

We further confirmed that *rdog* is upregulated in response to zinc supplementation using a graded series of ZnCl_2_ to supplement the culture media of wild-type S2R+ cells followed by qPCR, as shown in Figure 3. As expected, under ZnCl_2_-supplemented conditions, levels of the metallothionein-encoding gene *MtnA* are upregulated and levels of the zinc importer-encoding gene *foi* are downregulated. Under the same conditions, the levels of *rdog* are upregulated (Figure 3A). Based on the identification of *rdog* in the ZnCl_2_- but not the MnCl_2_-supplemented screen and transcriptomics data sets, we suspected that the effect was zinc-specific, rather than being attributable to the chloride ion. To further test this experimentally, we performed qPCR analysis on wild-type S2R+ cells supplemented with ZnSO_4_. The trends for control and *rdog* transcript levels were similar to those found for ZnCl_2_ (Figure 3B), demonstrating that *rdog* expression is upregulated by zinc in *Drosophila* S2R+ cells.

### Analysis of parallel studies using manganese chloride

As mentioned, we performed the RNAi screens and transcriptomics studies with MnCl_2_ in parallel to help distinguish zinc-specific factors from general factors, and to provide an additional metal intoxication-related data resource. For the MnCl_2_ toxicity screen, enrichment among genes conferring higher ATP levels upon knockdown is driven by the presence of multiple components of the vacuolar H+ ATP transport machinery (Figure 1B and Table 2). This suggests the possible relevance of proton transport to manganese-induced cell death or another related process. Several tetraspanin family proteins are also hits in the manganese screen. This is intriguing, as three tetraspanin family proteins were detected as coregulated by *trans*-eQTLs following feeding of flies with lead (Pb) (Ruden *et al.* 2009), suggesting the possibility of a general role for tetraspanin family proteins in detection of, or responses to, metals or metal-induced stress. Enrichment analysis of genes conferring lower ATP levels in the MnCl_2_ screen points to the relevance of mitochondria. With regards to transcriptomics analysis, we found that the results for MnCl_2_-treated samples were more useful as a comparison set for ZnCl_2_ treatment than for the detection of Mn-specific factors. Overall, the fold-change values were modest and a relatively small number of genes surpassed the cutoff values; for example, only 23 genes were downregulated and 33 upregulated in MnCl_2_-treated wild-type cells (Table 2). Nevertheless, overlap between these and the zinc treatment group was observed (Figure 2, Figure 3, and File S2), suggesting that the manganese treatment group provides a useful filter for further refinement of a list of candidate zinc-related factors.

### Conclusions

The functional genomics and transcriptomics data sets described here provide a genome-scale resource for the study of zinc biology in *Drosophila*. Despite the fact that we performed the RNAi screens under high-metal supplementation conditions, we were able to identify factors known to be relevant to zinc homeostasis at physiological levels (*i.e.*, *foi* and *CG32000*). This is consistent with known overlap between metal homeostasis and detoxification genes, and suggests the validity of the approach. In addition, despite assay bias, we were able to detect one high-confidence gene, *rdog*, for which knockdown results in lower ATP values as compared with the internal control. We further found that *rdog*, an ortholog of yeast YCF1, is upregulated in genetically zinc-sensitized cells following mild zinc supplementation. Identification of *rdog* in the cell-based screen, as well as identification in the transcriptomics data of *rdog*, *Gclc*, and genes encoding GSH S-transferase family proteins (Saisawang *et al.* 2012), supports the idea that GSH is relevant to zinc detoxification in *Drosophila*. Moreover, the observation that another gene encoding ABCC family member *CG7627* was also identified as a zinc sensitivity screen hit in this work, together with the fact that a third *Drosophila* ABCC family member, *dMRP*, has previously been implicated in methylmercury toxicity (Prince *et al.* 2014), suggest a general role for ABCC-type transporters in metal detoxification in *Drosophila*. Altogether, we expect that the ‘omics data presented here will guide further research into the mechanisms underlying metal homeostasis and detoxification in *Drosophila* and other systems. For example, the data provide a focused set of candidates for *in vivo* analyses of wild-type and genetically metal-sensitized flies under normal or metal-supplemented conditions, as well as for *in vivo* analyses in fly models of human diseases such as diabetes or neurodegeneration.

## 

## Supplementary Material

Supplemental material is available online at www.g3journal.org/lookup/suppl/doi:10.1534/g3.117.300447/-/DC1.
