# Supplementary material for: Zinc Detoxification: A Functional Genomics and Transcriptomics Analysis in Drosophila melanogaster Cultured Cells
Source: G3 (Bethesda). 2017 Dec 9;8(2):631–41. doi: 10.1534/g3.117.300447 (PMC5919732; doi:10.1534/g3.117.300447)
Supplement: Supplementary file 1 [file 631TableS1.pdf]

Table S1. Mouse inbred strains with CRISPR/Cas9 generated deletions within the *Slc25a13* gene. The deletions were made to test for their potential enhancer effects on *Dlx5* activity and inner ear development and function.

Deletion name	Deletion size (bp)	GRCm38 Chromosome 6 coordinates of deletion (bp)	Strain name	JAX Stock #
hs1642	440	6,163,224 - 6,163,663	C57BL/6J-*Slc25a13*^{em8Mvw}/Mvw	29894
hs2313 (eDlx#23)	455	6,058,558 - 6,059,012	C57BL/6J-*Slc25a13*^{em9Mvw}/Mvw	29895
Line 6	25,428*	6,141,226 - 6,166,653	C57BL/6J-*Slc25a13*^{em4Mvw}/Mvw	30007
Line 12	25,971	6,141,223 - 6,167,193	C57BL/6J-*Slc25a13e*^{m5Mvw}/Mvw	30008
Line 54	25,420*	6,141,226 - 6,166,645	C57BL/6J-*Slc25a13*^{em7Mvw}/Mvw	30010

* The Line 6 deletion includes a 3 bp insertion, and the Line 54 deletion includes a 2 bp insertion.

The individual sequences for each target and the PCR primers used to identify the alleles are as follows (all shown 5'-3').

hs1642 – sgRNA recognition regions: GATATGAATGTGAGATTT, GATGGGACACTTGTTTACAT; PCR and sequencing primers: TCTGTTTCCTCACTCAGCTTTAC, CTCTGTTTACCTGGGATGCTATG.

hs2313 – sgRNA recognition regions: GATAAAGATGTCAGAGAC, GCCTGGCTGTGCATGGAA; PCR and sequencing primers: GATGCTGGCCATAGTATCCTTAC, TGCTCAAGTTCCATGGTATTACT.

25 kb deletions (Lines 6, 12, and 54) – sgRNA recognition regions: GCTGTGCGACACCTCTTT, GAGCAGGTCATTCGCCTA; PCR and sequencing primers: GGCCCACGTGTAATGGTAAT, GGATAGGGTGCTATGTCTGTAATG.

Bridging oligonucleotide (used in the generation of Line 6 and 30008 only):

CTTAAGATGGACAAAAGATAAAAGGACAGTTTTCAAATGGGAAAAAATAGAAACTAAGGAGACATTTTTCTAAAATAAAAATGGCCCACAAGTACCTAAACTAAGGTCTCAGAGGTTTTAAGAGACCTAGTAAAAGACAAATGCTGTGTCAGCCACCACTACTGTTCCTTCCACTATGCAATGCTTTTCGTATGAGAAAG.
